# Unfair teachers, unhappy students: longitudinal associations of perceived teacher relational unfairness with adolescent peer aggression and school satisfaction

**DOI:** 10.3389/fpsyg.2024.1321050

**Published:** 2024-04-19

**Authors:** Gianluca Gini, Federica Angelini, Tiziana Pozzoli

**Affiliations:** Department of Developmental Psychology and Socialization, University of Padua, Padua, Italy

**Keywords:** perceived teacher unfairness, reactive aggression, proactive aggression, school satisfaction, teacher injustice

## Abstract

**Introduction:**

Teacher relational unfairness is a significant risk factor for students’ physical and mental well-being, especially during adolescence. However, school psychology research has not yet fully analyzed the links between teacher unfairness and important indicators of school experience and wellbeing, including peer aggression and school satisfaction. Even less evidence does exist with longitudinal, multilevel data.

**Methods:**

The present study tested the prospective relations between Fall perceived teacher unfairness and Spring reactive and proactive aggression, and school satisfaction. At T1, participants were 1,299 students (48.3% girls, mean age = 13.6 years, SD = 1.1) attending 67 classrooms in Italian public schools, whereas 1,227 students participated in the second wave 6 months later.

**Results:**

Multilevel regressions showed that, at the individual level, T1 perceived teacher unfairness positively predicted T2 reactive and proactive aggression, and negatively predicted school satisfaction. At the class-level, T1 class teacher unfairness explained between-class variability in T2 school satisfaction, but not variability in peer aggression.

**Discussion:**

The findings expand current knowledge about the role of teacher unfairness with the classroom and have implications for interventions at school.

## 1 Introduction

Teacher unfairness refers to students’ perceptions of being treated unfairly by their teachers. Relational (or interpersonal) unfairness, specifically, pertains to the evaluation of how individuals are treated in terms of fairness, honesty, and respect within their interpersonal relationships (Colquitt, 2001; Lenzi et al., 2013; Rasooli et al., 2019). This represents a significant, yet relatively understudied component of negative teacher-student relationships within the classroom microsystem, which can be a potentially anxiety-inducing situation able to influence students’ behavior, well-being, and adjustment (Bronfenbrenner, 1979; Swearer and Hymel, 2015).

When individuals perceive fairness in their treatment, they tend to view those in authority as more reliable and trustworthy; moreover, they experience an enhanced sense of self-worth and have a greater feeling of belonging and self-esteem (Tyler and Smith, 1999; Cropanzano et al., 2001). In the classroom, teacher fairness and respect for students contribute to a better relational climate and reduce negative behaviors (Murdock, 1999). Conversely, unfair treatment more likely leads to emotions such as anger, frustration, or anxiety (Roeser et al., 1998).

With few exceptions, most of the knowledge about the relation of unfair treatment with individual adjustment derives from social/organizational psychology and adult samples. However, in the last decade, consistent calls for explicit investigations of fairness issues within classroom contexts have been advanced (Kazemi, 2016; Sabbagh and Resh, 2016; Rasooli et al., 2019). While school-based research has focused more on distributive and procedural fairness, especially in relation with academic motivation, engagement, and performance (Rasooli et al., 2019), interpersonal (or relational) fairness has been much less analyzed. In fact, a few studies so far have addressed the association between teacher relational unfairness and physical/mental health outcomes among adolescents in the school context (e.g., Santinello et al., 2009; Gini et al., 2018). Even less studies have tried to answer whether higher levels of teacher unfairness might explain adolescent students’ poor school adjustment, in terms of peer aggression and school satisfaction. Adopting a longitudinal, multilevel approach, the current study aimed at investigating this research question over the course of one school year. Specifically, it was tested whether perceived teacher unfairness in the Fall contributed to explain students’ reactive and proactive aggression and school satisfaction in the Spring, after controlling for the stability of the outcomes and for school stress related to academic demands. Moreover, beyond individual-level effects, it was analyzed the potential role of teacher unfairness at the class-level, investigating whether, on average, higher levels of teacher unfairness corresponded to increased peer aggression and reduced school satisfaction in school classes. The focus on both levels is an important novelty of this study. The study involved adolescent students because research suggested that being treated unfairly by teachers is a more frequent school stressor among adolescents compared to children (Hjern et al., 2008) and because unfair treatment can be particularly destructive during early/middle adolescence due to adolescents’ heightened sensitivity to social comparisons (Osterman, 2000).

### 1.1 Perceived teacher unfairness and peer aggression

One potential negative correlate of perceived teacher unfairness relevant for students’ school adjustment is aggressive behavior (e.g., Vieno et al., 2011; James et al., 2015). Even though many components of the classroom context might play a role in students’ aggressive behavior, one aspect that has received limited attention is how students perceive the fairness (or lack thereof) in their treatment by teachers. According to classic equity and social exchange theories (e.g., Adams, 1965) and the cognitive appraisal model of stress (Lazarus, 1966), the social/organizational literature about justice has reported that adults’ actions of relational unfairness can stimulate anger and aggressive behavior (e.g., Skarlicki and Folger, 1997). Moreover, consistent with the ecological systems theory (Bronfenbrenner, 1979), regular experiences of perceiving unfair treatment from teachers could potentially contribute to the propagation of social norms that tolerate disrespectful and dominating behaviors. Adolescents may view these behaviors as acceptable in the classroom context and adopt them accordingly. Ultimately, this could lead to imbalanced peer interactions characterized by dominance and aggression, or to the use of aggressive behavior as a means to address conflicting or frustrating situations among classmates. Furthermore, teachers’ unfair treatment can erode their authority legitimacy (Tyler and Lind, 1992), increasing the likelihood of student involvement in aggressive behavior as they imagine they will not face consequences (Santinello et al., 2011; Vieno et al., 2011).

According to a recent meta-analysis, “a particularly damaging link exists between teachers’ poor relationships with students and negative interactions in the peer context, at least as it concerns involvement in peer aggression and bullying” (Krause and Smith, 2022, p. 321). However, there have been limited studies that have specifically examined the impact of perceived teacher unfairness; these studies focused, mainly, on school bullying and employed cross-sectional designs. For example, in a large sample of early, Santinello et al. (2011) found that teacher unfairness was significantly associated with being a bully or a bully-victim, even after adjusting for several potential confounding factors, including age, sex, socio-economic status, empowerment with friends, school achievement, and trust in people. Another study (Lenzi et al., 2014) found that the association between perceived teacher unfairness and school bullying was partially mediated by endorsement of instrumental goals. According to the social information processing model of aggression proposed by Crick and Dodge (1994), this finding aligns with the notion that perceived unfairness from teachers might serve as a potential mechanism affecting students’ socio-moral cognition and promoting the occurrence of bullying behaviors. As argued by Arsenio and Gold (2006), socio-moral cognitions, including the bias of valuing instrumental goals more than relational ones, may partly stem from unfairness experienced in different social contexts, including the classroom. Adolescents who believe they are being treated unjustly by their teachers can potentially cultivate a cynical and pessimistic perspective on the concept of morality as a form of authority. This perception may subsequently influence their behavior when interacting with their peers.

However, bullying is not the only form of peer aggression at school and research on the role of teacher unfairness in broader adolescents’ aggression conducts is necessary, especially with longitudinal data. Bullying is the most common form of proactive aggression, while many students also rely on reactive aggression to deal with peer conflicts (Little et al., 2003). These two forms of aggression certainly overlap to some degrees, but they are also conceptually distinct and can have different correlates (Polman et al., 2007). Little research, however, has explicitly focused on the influence of teacher unfairness for reactive and proactive aggression separately. To the best of our knowledge, only one study conducted with a large sample of Chinese adolescents (Ren et al., 2023) has provided preliminary results on the association between perceived teacher unfairness and reactive and proactive aggression. Even though it was not the main aim of that study, the authors found positive bivariate correlations between teacher unfairness and both reactive and proactive aggression, both concurrently and longitudinally after 6 months. In line with this, the first aim of the present study was to test the longitudinal relations between individual student’ perceived teacher unfairness and both reactive and proactive aggression in a sample of adolescents.

### 1.2 Perceived teacher unfairness and school satisfaction

Teacher unfairness toward students can also influence their motivation, satisfaction with, and adjustment at school (Stipek et al., 1998; Ripski and Gregory, 2009; Bayram Özdemir and Özdemir, 2020). For example, it has been shown that perceived unfair treatment is associated with lower adolescents’ academic motivation and perceived academic value (Roeser et al., 1998), whereas teacher fairness is linked to students’ school engagement (Danielsen et al., 2010). Students also tend to be more satisfied when they belong to classrooms where teachers treat them fairly (Samdal et al., 1998). Low school satisfaction, indeed, seems to be one of the most important negative correlates of teacher unfairness. For instance, in a recent investigation with adolescents, Gini et al. (2018) have found that perceived teacher unfairness was associated with poorer psychological and physical health, lower satisfaction with school and friends at school, and lower sense of safety. Among these effects, the relation between teacher unfairness and school satisfaction was the strongest one. Interestingly, perceived teacher unfairness uniquely contributed to explain adolescent’ school satisfaction even after controlling for another important school stressor, that is, peer victimization (Gini et al., 2018). However, because prior studies have employed cross-sectional designs, we aimed to expand previous findings by testing whether the negative role of perceived teacher unfairness in explaining students’ school satisfaction is confirmed longitudinally, over the course of 6 months. Moreover, we wanted to make sure that students reported lower school satisfaction was not due to other related academic reasons. Therefore, we controlled for the potential effect of school stress, in terms, for example, of high pace of schoolwork (Hjern et al., 2008).

### 1.3 Sex differences

Literature findings on sex differences vary according to the specific variable we take into consideration. Sex differences consistently emerge for mean levels of peer aggression, with males reporting higher levels of aggressive behavior than females (Card et al., 2008); to a lesser extent, sex seems to play a role also in school satisfaction, even though results do not consistently favor males or females (Löfstedt et al., 2020). To the best of our understanding, only two research studies have specifically examined gender disparities when it comes to the impact of perceived teacher unfairness on individual outcomes. One study (Lenzi et al., 2014) did not report significant sex differences in the cross-sectional association between perceived teacher unfairness and bullying. In the second study (Gini et al., 2018), adolescent girls showed stronger links between perceived teacher unfairness and satisfaction with school. In the current work we explored whether links between the constructs of this study were different for males and females.

### 1.4 Perceived teacher unfairness at the class-level

While research at the class-level on broader concepts of negative teacher-student relationships—which sometimes include, but are not limited to perceived teacher unfairness—exists (e.g., Thornberg et al., 2018; Ten Bokkel et al., 2023), most of the literature focused on relational unfairness has restricted the analysis to the individual students’ perceptions and how they relate to the outcomes of interest. However, when we take into account the classroom setting, we come across a significant concern regarding fairness, specifically the environment in which a judgment is formed. In the social/organizational literature, for example, fairness context—the average of individual perceptions of fairness within a group—has been found to predict satisfaction above and beyond individual-level perceptions of fairness (Mossholder et al., 1998; Naumann and Bennett, 2000). The given evidence indicates that certain attributes or traits of the group, which can be considered as a representation of the teacher’s fairness, might have a connection to the variations in overall satisfaction among different groups (Wendorf and Alexander, 2005).

Apart from a few instances (e.g., Vieno et al., 2011), there has been limited exploration into the impact of class-level perceptions of relational fairness on students’ behavior or level of satisfaction. Another important limitation of the current literature of teacher relational unfairness is therefore lack of systematic investigations of both individual-level and class-level longitudinal effects. In this context, it is crucial to grasp the distinctive traits of the Italian educational system and their potential influence on how adolescents perceive unfair treatment by teachers. Students stay in the same classroom alongside a fixed group of classmates and the same teachers throughout the entire school year (and usually for more than 1 year). In the context of a classroom, the interactions between teachers and students play a significant role in shaping the overall environment. These interactions are particularly crucial and influential in comparison to other countries. Hence, an essential objective of this study was to explore the connection between unfair treatment experienced in the classroom and the subsequent manifestation of aggressive behavior and satisfaction levels of adolescents in school.

### 1.5 The current study

The objective of this study was to examine the relationship between fairness exhibited by teachers at both the individual and class levels and peer aggression and school satisfaction among a group of adolescents throughout a school year. At the individual level, it was hypothesized that, after controlling for the stability of the same behavior, for the other outcomes, and for academic school stress, perceived teacher unfairness measured in the Fall would be positively associated with peer aggression in the Spring. In order to contribute to the current body of knowledge, this study incorporated both reactive and proactive aggression as potential outcomes. Due to lack of previous data, it is uncertain whether perceived teacher unfairness might be a stronger risk factor for one type of aggression than another, or whether it is a similar risk factor for all types of aggressive behavior. Drawing upon the restricted empirical evidence at hand, and theories of unfairness described above, it was expected to find comparable effects of perceived teacher unfairness on both forms of aggression over a period of 6 months. Moreover, we expected higher levels of perceived teacher unfairness to be associated with lower school satisfaction 6 months later. These hypotheses were based both on the theories and the previous cross-sectional findings reviewed above.

Moreover, even though a thorough analysis of sex differences was not within the scope of the current study, in the current sample we explored whether links between the constructs of this study were different for males and females. That is, we tested for a possible moderation effect of sex on the longitudinal associations between perceived teacher unfairness and the three outcomes.

Regarding the class-level analysis, a positive longitudinal association was hypothesized between class teacher unfairness and both reactive and proactive aggression. It was anticipated that there would be a higher likelihood of aggressive behavior in the Spring in school classrooms where teachers were perceived to be more unfair during the preceding Fall. Similarly, we expected that between-class variability of school satisfaction would be significantly explained by class levels of teacher unfairness, so that students would report on average lower school satisfaction if they belong to classrooms with higher levels of perceived teacher unfairness. At both levels of analysis, the hypothesized effect of perceived teacher unfairness was tested controlling for the individual-level and class-level stability of the outcomes, and for school stress.

Finally, it was examined whether there was between-class variability in the associations between perceived teacher unfairness and T2 outcomes, and whether any variation could be explained by class teacher unfairness (i.e., cross-level interaction).

## 2 Materials and methods

### 2.1 Sample

The data used in this study were extracted from a larger dataset of a longitudinal project examining the social-cognitive factors associated with aggressive behavior in adolescents. A portion of this dataset was previously utilized in another study on the moral predictors of aggressive behavior (Gini et al., 2022). Although the sample is the same, there is minimal overlap between the data used in the previous study and the current study, with only age, sex, and reactive and proactive aggression being common variables, serving different research purposes. The data on teacher unfairness, school satisfaction, and school stress have never been used in previously published manuscript. A total of 67 classes from 9 public schools in urban and suburban areas of Northern Italy participated, comprising students in grades 7th to 10th (typically aged 12 when entering grade 7th in Italy). The average class size was 20.1 students.

The first wave of data collection occurred in December 2017, approximately 3 months after the beginning of the school year. At that time, 1,299 students (48.3% girls, mean age = 13.6 years, *SD* = 1.1) completed the study measures. For the second wave (May 2018, near the end of the school year), 1,227 students (48.7% girls) participated, resulting in a retention rate of 94%. Out of these participants, 6 students did not respond to the reactive-proactive aggression scale, and 3 students did not complete the items about school satisfaction. Attrition analyses were conducted to examine differences between students who participated in both waves and those who did not. Findings indicated no differential attrition based on gender (*χ*^2^ = 0.70, *p* = 0.71) or no significant differences in reactive and proactive aggression and school stress. However, students who did not participate in the second wave were slightly older than those who took part in both waves (*M*_age_ = 14.18 vs. *M*_age_ = 13.60*; t* = 5.71, *p* < 0.001); they also reported lower school satisfaction (*M* = 2.93 vs. *M* = 3.17; *t* = 3.88, *p* < 0.001) and higher perceived teacher unfairness (*M* = 2.41 vs. *M* = 2.18; *t* = 3.15, *p* < 0.001).

Regarding ethnic/cultural background, the majority of participants (88.9%) had both parents born in Italy, aligning with national statistics on the Italian student population (MIUR, 2019). Conversely, 11.1% of students had one or both parents born in foreign countries. Socioeconomic background was assessed using the Family Affluence Scale III (Torsheim et al., 2016), a validated measure of family socioeconomic status (SES). Most participants came from medium- and high-class families (low FAS: 7.2%; medium FAS: 59.7%; high FAS: 33.1%).

### 2.2 Measures

#### 2.2.1 Perceived teacher unfairness

To assess perceived teacher unfairness, a 6-item scale was employed (Gini et al., 2018). This scale measured students’ perceptions regarding the extent to which they were treated fairly and respectfully by their teachers. Sample items included “My teachers treat me fairly” (reverse scored) and “I am treated too severely by my teachers.” Participants indicated their agreement on a 5-point scale, ranging from 1 (completely disagree) to 5 (completely agree). Before computing participants’ scores, positively worded items were reverse scored to ensure that higher scores reflected greater perceived teacher unfairness. Previous studies involving Italian adolescents (Gini et al., 2018) have demonstrated good psychometric properties, including good test–retest reliability (*r* = 0.67). In this sample, the scale confirmed a good factorial structure (CFA: *χ*^2^ = 2.10, *p* = 0.91, CFI = 1, RMSEA = 0.00, SRMR =0.004). The internal consistency for the current sample was Cronbach’s *α* = 0.76 (95% CI = 0.74–0.78), McDonald’s ω = 0.73.

#### 2.2.2 Reactive and proactive aggression (T1 and T2)

At both waves of the study, participants’ reactive and proactive aggressive behavior was measured using a 24-item scale (Little et al., 2003). Reactive aggression was assessed with 12 items describing reactions to being hurt or upset by others (e.g., “When I’m hurt by someone, I often fight back;” “If others upset or hurt me, I often tell my friends to stop liking them”), while proactive aggression was assessed with 12 items measuring aggressive actions taken to achieve personal goals (e.g., “I often start fights to get what I want,” “I often tell my friends to stop liking someone to get what I want”). Participants rated the frequency of their aggressive behavior on a 6-point scale ranging from 1 (not at all) to 6 (very much).

This scale has been previously utilized with Italian adolescents and has demonstrated good psychometric properties (e.g., Gini et al., 2015). Longitudinal scalar invariance in this sample was confirmed as reported in more details in the Results section. Accordingly, for each participant, responses to relevant items were averaged to obtain scores for reactive aggression and proactive aggression at T1 (reactive aggression: Cronbach’s α = 0.89, 95% CI = 0.98–0.90, McDonald’s *ω* = 0.93; proactive aggression: *α* = 0.95, 95% CI = 0.94–0.95, *ω* = 0.97) and T2 (reactive aggression: *α* = 0.89, 95% CI = 0.88–0.90, *ω* = 0.93; proactive aggression: *α* = 0.96, 95% CI = 0.95–0.96, *ω* = 0.98).

#### 2.2.3 School satisfaction (T1 and T2)

Participants’ school satisfaction was measured using a subscale of the Multidimensional Students’ Life Satisfaction Scale (Huebner, 1994; Gilman et al., 2000). This subscale consisted of 8 items measuring adolescents’ satisfaction specifically related to school (e.g., “I look forward to going to school,” “I like being in school”). At both waves, participants provided answers on a scale ranging from 1 (completely disagree) to 5 (completely agree). Negatively keyed items were reversed scored ensuring that higher scores indicated greater levels of satisfaction. Previous studies involving Italian adolescents (Gini et al., 2018) have demonstrated good psychometric properties, including satisfactory test–retest reliability (*r* = 0.77). Longitudinal scalar invariance in this sample was confirmed as reported in more details in the Results section. The internal consistency of the scores in this sample was *α* = 0.72 (95% CI = 0.70–0.75), *ω* = 0.84 at T1 and *α* = 0.74 (95% CI = 0.72–0.76), *ω* = 0.85 at T2.

#### 2.2.4 School stress

Students’ feelings of stress at school were measured using a 4-item scale adapted from previous studies (Byrne et al., 2007; Hjern et al., 2008). Participants were asked to rate how frequently in the last 3 months they thought that, for example, the pace of the schoolwork was too high or that there were too many class assignments and oral tests. Answers were provided on a 5-point scale, ranging from 1 (never) to 5 (10 times or more). The scale showed good factorial structure (CFA: *χ*^2^ = 3.94, *p* = 0.19, CFI = 0.99, RMSEA = 0.022, SRMR = 0.008). The internal consistency for the current sample was *α* = 0.77 (95% CI = 0.75–0.77, *ω* = 0.78).

#### 2.2.5 Class-level variables

To test one of our main hypothesis about the role of perceived teacher unfairness at the class-level and to account for the stability of the outcomes within each classroom, aggregated scores of each variable were created by averaging the individual scores among classmates, aligning with previous research on class norms and characteristics (e.g., Salmivalli and Voeten, 2004; Busching and Krahé, 2020; Szumski et al., 2020).

### 2.3 Procedure

First, school principals granted authorization for the classes to participate in the study. Parents of the students then provided active consent by signing a letter that informed them about the study and its objectives. Less than 10% of students in the participating classrooms did not receive parental consent. Assent for participation was also obtained from adolescents with parental consent; no one refused to participate. Data collection occurred twice within one school year, where participants completed a web-based questionnaire during a regular school hour. An anonymized alphanumeric code was used to match T1 and T2 data. A graduate research assistant was present during data collection and assured participants that their responses would remain confidential. Participants were encouraged to seek assistance if needed. At the end of data collection, any questions regarding the questionnaires or the overall aims of the project were addressed. The study protocol was approved by the local Ethics Committee for Research in Psychology (protocol #1157/2012).

### 2.4 Data analyses

Missing data were minimal, with only a small number of students having failed to complete the full list of items. To handle missing data, full information maximum likelihood (FIML) estimation (Enders and Bandalos, 2001) in Mplus was used, so that all available information was used in the model estimation.

As a preliminary step, longitudinal confirmatory factor analyses were conducted on the aggression scores and on school satisfaction at both waves to check for longitudinal invariance. A three-factor model (reactive aggression, proactive aggression, and school satisfaction) was tested. The assumption of invariance was evaluated based on change in value of fit indices (i.e., ΔCFI, ΔRMSEA, and ΔSRMR). Negligible change, that is, a ΔCFI smaller than 0.01 and a change smaller than 0.015 in RMSEA and SRMR, was considered indicative of invariance (e.g., Cheung and Rensvold, 2002; Chen, 2007). Subsequently, multivariate multilevel modeling was performed in Mplus 8.3 (Muthén and Muthén, 1998–2017). The three simultaneous dependent variables were reactive and proactive aggression and school satisfaction in Spring (T2). In this way, we took into account the intercorrelation between the outcomes, while testing the relative strength of each of the respective predictors. At the individual-level, sex (0 = males, 1 = females), age, fall levels of reactive and proactive aggression, of school satisfaction, and of school stress were entered as control variables. Perceived teacher unfairness at T1 was the key predictor. To enhance interpretability, individual-level variables, except sex, were group-mean centered. In addition to main effects, the two-way interaction between perceived teacher unfairness and sex was entered. At level 2, grade, T1 class-level aggression scores, and class-level school satisfaction and school stress were entered as control variables; T1 class-level perceived teacher unfairness was entered as main contextual predictor. Variables at the class-level were grand-mean centered.

Finally, it was examined whether there was between-classroom variability in the associations between perceived teacher unfairness and T2 aggression and school satisfaction (i.e., random slopes). In case a significant random slope emerged, cross-level interaction would be tested to check if a level-2 variable could explain the slope variability.

## 3 Results

### 3.1 Descriptive statistics and correlations

Tables 1, 2 report descriptive statistics and correlations at the individual-level and the class-level, respectively. As expected, both types of aggressive behavior and school satisfaction showed significant stability across the school year. Moreover, perceived teacher unfairness was positively, but weakly associated with both types of aggression, and negatively and strongly associated with school satisfaction, both at the individual and the class-level.

**Table 1:** Means, standard deviations and correlations among variables at the individual level.

	*M*	*SD*	1	2	3	4	5	6	7
1. Reactive aggression T1	2.03	0.86	–						
2. Reactive aggression T2	2.03	0.86	0.57	–					
3. Proactive aggression T1	1.45	0.75	0.73	0.42	–				
4. Proactive aggression T2	1.48	0.82	0.41	0.76	0.46	–			
5. School satisfaction T1	3.15	0.66	−0.18	−0.21	−0.17	−0.20	–		
6. School satisfaction T2	3.09	0.66	−0.20	−0.22	−0.15	−0.15	0.68	–	
7. Perceived teacher unfairness T1	2.21	0.76	0.20	0.23	0.20	0.23	−0.51	−0.49	–
8. School stress T1	2.51	0.96	0.22	0.18	0.20	0.12	−0.22	−0.22	0.22

All *p*s < 0.001.

**Table 2:** Means, standard deviations and correlations among variables at the class level.

	*M*	*SD*	1	2	3	4	5	6	7
1. Class-level reactive aggression T1	2.04	0.27	–						
2. Class-level reactive aggression T2	2.03	0.30	0.62^***^	–					
3. Class-level proactive aggression T1	1.45	0.25	0.74^***^	0.52^***^	–				
4. Class-level proactive aggression T2	1.48	0.34	0.47^***^	0.87^***^	0.52^***^	–			
5. Class-level school satisfaction T1	3.15	0.37	−0.25^***^	−0.28^***^	−0.14^***^	−0.26^***^	–		
6. Class-level school satisfaction T2	3.08	0.36	−0.24^***^	−0.25^***^	−0.09^***^	−0.20^***^	0.87^***^	–	
7. Class-level teacher unfairness T1	2.21	0.44	0.18^***^	0.15^***^	0.08^**^	0.07^*^	−0.83^***^	−0.81^***^	–
8. Class-level school stress T1	2.51	0.35	0.11^***^	0.11^***^	0.20^***^	0.06^*^	−0.05	−0.05	0.02

^*^p < 0.05, ^**^p < 0.01, ^***^p < 0.001.

### 3.2 Multilevel analyses

As preliminary analysis, we performed longitudinal confirmatory factor analyses on the aggression scores and on school satisfaction at both waves. First, test of configural invariance in the two waves yielded an acceptable fit: (*χ*^2^ = 12,064, *p* < 0.001, CFI = 0.914, RMSEA = 0.062, SRMR =0.08). Second, constraining all loadings to equality across waves (i.e., metric invariance) did not lead to reduction in model fit (ΔCFI = −0.008; ΔRMSEA = −0.003; ΔSRMR = 0.000). Finally, scalar invariance was also checked (ΔCFI = 0.001; ΔRMSEA = −0.003; ΔSRMR = 0.000), confirming longitudinal invariance of the three scores.

We then estimated an unconditional model to calculate how much variance of T2 reactive and proactive aggression and T2 school satisfaction existed at the individual- and class-level. Within- and between-level variance estimates were the following: 0.685 and 0.047 for reactive aggression, 0.591 and 0.078 for proactive aggression, and 0.329 and 0.106 for school satisfaction. The intraclass correlation coefficients (ICC) therefore indicated that 6.3% of the variation of reactive aggression, 11.6% of the variation of proactive aggression, and 23.9% of the variation of school satisfaction were due to differences between classes. Based on the ICC values and an average classroom size of 20, estimated design effects were 2.20 for reactive aggression, 3.20 for proactive aggression, and 5.54 for school satisfaction, further supporting the appropriateness of adopting a multilevel analytical framework. Moreover, the ICC for teacher unfairness was 0.31, also confirming the non-independence of the unfairness perceptions within the classes and the meaningfulness of computing a class-level teacher unfairness score.

Results of the full multilevel model are reported in Table 3. At the individual level, the model explained 34.6% of variance for reactive aggression, 27% of variance for proactive aggression and 36.3% of variance for school satisfaction. At the class level, the variance explained was 61.2% for reactive aggression, 40% for proactive aggression, and 90.3% for school satisfaction. For the sake of simplicity, the findings for the three outcome variables are summarized separately in the following paragraphs.

**Table 3:** Multivariate multilevel modeling predicting T2 aggression and school satisfaction.

	Reactive aggression (T2)				Proactive aggression (T2)				School satisfaction (T2)			
	*b*	*SE*	*z*	*p*	*b*	*SE*	*z*	*p*	*b*	*SE*	*z*	*p*
*Individual level (T1)*												
Age	0.059	0.07	0.913	0.361	0.086	0.07	1.24	0.214	0.027	0.03	0.84	0.401
Sex (0 = M, 1 = F)	−0.213	0.05	−4.42	<0.001	−0.285	0.06	−5.04	<0.001	−0.011	0.04	−0.32	0.752
Reactive aggression	0.527	0.04	12.07	<0.001	0.080	0.04	1.89	0.059	−0.078	0.03	−2.74	0.006
Proactive aggression	−0.063	0.06	−1.16	0.252	0.332	0.06	6.01	<0.001	0.048	0.03	1.66	0.097
School satisfaction	−0.078	0.05	−1.71	0.088	−0.044	0.05	−0.93	0.353	0.522	0.03	17.30	<0.001
School stress	0.035	0.03	1.36	0.175	−0.002	0.03	−0.07	0.943	−0.030	0.01	−2.12	0.034
Perceived teacher unfairness	0.179	0.07	−2.58	0.010	0.234	0.08	3.11	0.002	−0.129	0.03	−4.05	<0.001
Sex * Unfairness	−0.146	0.10	−1.55	0.122	−0.183	0.08	−2.17	0.030	0.004	0.05	0.08	0.933
*Class level (T1)*												
Grade	0.030	0.03	1.19	0.233	0.035	0.03	1.04	0.298	−0.012	0.02	−0.63	0.527
Class-level reactive aggression	0.583	0.15	3.94	<0.001	0.225	0.19	1.17	0.241	−0.110	0.09	−1.20	0.230
Class-level proactive aggression	−0.005	0.17	−0.03	0.975	0.315	0.22	1.42	0.156	0.115	0.10	1.17	0.244
Class-level school satisfaction	−0.136	0.16	−0.83	0.409	−0.323	0.21	−1.51	0.131	0.595	0.09	6.43	<0.001
Class-level school stress	0.016	0.07	0.22	0.825	−0.022	0.09	−0.24	0.813	−0.076	0.04	−1.90	0.058
Class-level teacher unfairness	−0.092	0.12	−0.77	0.443	−0.242	0.15	−1.67	0.096	−0.206	0.07	−2.93	0.003
*R^2^*_individual level_	0.346				0.270				0.363			
*R^2^*_class level_	0.612				0.400				0.903			

*b* indicates unstandardized estimates from multilevel modeling.

#### 3.2.1 Reactive aggression

At the individual level, reactive aggression was found to be moderately stable over the course of the school year (*b* = 0.527, *SE* = 0.04, *p* < 0.001) and, as expected, more frequent among males (*b* = −0.213, *SE* = 0.05, *p* < 0.001). After taking all the other variables into account, as hypothesized, higher levels of perceived teacher unfairness at T1 positively predicted T2 students’ reactive aggression (*b* = 0.179, *SE* = 0.07, *p* = 0.010). At the class-level, after controlling for the stability of the aggressive behavior, no significant predictor emerged.

#### 3.2.2 Proactive aggression

Regarding proactive aggression, the analysis at the individual level yielded one significant main effect and an interaction, beyond the expected effects of T1 proactive aggression (*b* = 0.332, *SE* = 0.06, *p* < 0.001) and sex (*b* = −0.285, *SE* = 0.06, *p* < 0.001). Consistent with our hypothesis, students who perceived higher teacher unfairness at T1 reported more proactive aggression at T2 (*b* = 0.234, *SE* = 0.08, *p* = 0.002). This main effect was qualified by a significant interaction between perceived teacher unfairness and sex. Simple slope analysis revealed a positive association between perceived teacher unfairness and T2 proactive aggression for male adolescents (*b* = 0.242, *p* = 0.002), but not for female adolescents (*b* = 0.012, *p* = 0.82).

At the class level, similar to the findings for reactive aggression, none of the predictors significantly explained between-class variation in scores of proactive aggression at the end of the school year.

#### 3.2.3 School satisfaction

Results about school satisfaction showed that reporting more reactive aggression (*b* = −0.078, *SE* = 0.03, *p* = 0.006) and more school stress (*b* = −0.03, *SE* = 0.01, *p* = 0.034) at T1 was associated with lower school satisfaction at the end of the school year. Regarding our study hypothesis, perceived teacher unfairness was a significant negative predictor of school satisfaction (*b* = −0.129, *SE* = 0.03, *p* < 0.001). At the class-level, between-class variability in school satisfaction was significantly explained by class-level teacher unfairness (*b* = −0.206, *SE* = 0.07, *p* = 0.003), even after controlling for the stability of class-level school satisfaction and for school stress.

#### 3.2.4 Cross-level interactions

As a final step, it was explored whether the slopes of the associations between perceived teacher unfairness and T2 aggressive behavior and school satisfaction varied across classrooms. However, no significant cross-level interactions emerged. Therefore, in the interest of parsimony, cross-level interactions were not included in the final model.

## 4 Discussion

As educators within the classroom, influential individuals, and catalysts for social development, teachers can shape how students interact with each other and adjust to school life. Within the broader literature on teacher-student relationships and classroom justice, the current study contributed to expand the still limited empirical evidence about teacher relational unfairness by testing its longitudinal associations with important indicators of students’ school adjustment and well-being, namely peer aggression and school satisfaction. Specifically, we included two types of aggressive behavior (i.e., reactive and proactive aggression), to further add to the current literature on teacher relational unfairness that has almost only focused on a specific subtype of aggressive behavior (i.e., bullying). The results have confirmed that when students perceive unfair treatment from their teachers, it can heighten the likelihood of them displaying aggressive behavior within the classroom. Additionally, this perception of unfair treatment can also result in lower levels of satisfaction among students with regard to their overall school experience.

### 4.1 Individual-level effects

First, findings from the multivariate multilevel modeling confirmed the hypothesis that perceived teacher unfairness at the individual-level significantly predicted the three outcomes of interest 6 months later. This result was robust after controlling for the stability of each outcome, for the other outcomes, and for academic school stress. In simple terms, students who reported higher levels of teacher relational unfairness in the Fall were more likely to report both reactive and proactive aggression in the Spring. Moreover, the higher the perception of being treated unfairly by teachers, the lower the school satisfaction at the end of the school year. These findings are consistent with the previous evidence reviewed above about the cross-sectional association of perceived teacher unfairness especially with bullying (Santinello et al., 2011; Lenzi et al., 2014), fighting (Vieno et al., 2011), and school satisfaction (Samdal et al., 1998; Gini et al., 2018) that guided our study hypotheses. Moreover, they expand the current knowledge in that the negative role of teacher unfairness emerged in a short-term longitudinal design, with data modeled at both the individual- and class-level, and after controlling for potential confounders. Although not conclusive, these findings are clearly important and call for further longitudinal investigations on the role of teacher relational unfairness in other important domains of students’ school life.

Theoretically, the current findings are consistent with, and give further support to, the predictions of the social ecological theory (Bronfenbrenner, 1979) and social-cognitive models of stress applied to the school context (Swearer and Hymel, 2015), which indicate perceiving unfairness from teachers within the classroom microsystem as a meaningful, negative experience able to influence adolescents’ well-being and school/social adjustment. Teachers have a significant role in managing the dynamics within the classroom when it comes to their interactions with students. They not only promote and reinforce positive conduct but also discourage any negative behavior. Moreover, teachers act as a mediator, fostering harmonious relationships among students within the class-group (Marengo et al., 2018). Positive relationships with students, marked by low conflict and high closeness, are linked to students’ better adjustment to the school context (Baker, 2006; Longobardi et al., 2019), greater academic commitment (Longobardi et al., 2016, 2019), and to prosocial behavior and reduced aggression (Jungert et al., 2016; Marengo et al., 2018). Conversely, low-quality relationships with students can undermine the teachers’ ability to perform these functions.

Interestingly, the main effect of perceived teacher unfairness for proactive aggression was moderated by sex, indicating that this positive association was apparent only for the male group. This result is quite unexpected, especially in light of what emerged in Lenzi et al. (2014) study that did not find evidence of sex differences in the association between perceived teacher unfairness and a specific type of proactive aggressive behavior (i.e., bullying). This different finding may be explained by the different design and model of analysis of our study, by sample differences, and other potential methodological differences, but it is currently impossible to draw conclusions. Sex is indeed often identified as a significant moderator in the peer aggression literature, even though it is not always easy to understand what really makes some associations stronger for one sex group compared to the other. In sum, although exploratory, it remains an interesting result and it suggests that further research is certainly warranted to better explore how and under what circumstances perceiving to be treated unfairly at school may be differentially important for females’ and males’ adjustment and well-being.

### 4.2 Class-level effects

Using the lens of the socio-ecological model, another aim of this study was to explore the classroom as an important context that influences students’ behavior and satisfaction. Regarding average differences of peer aggression at the class-level, after controlling for the stability of the aggressive behavior, no significant predictor emerged. This did not fully confirm what was found in the few previous studies that have analyzed—cross-sectionally—the role of class teacher unfairness in students’ bullying and violence (Vieno et al., 2011). The lack of statistically significant effects in this study may, of course, have different explanations. The first is the relative stability of the two class-level variables over the short period of time considered in our project. Moreover, even though there was a certain degree of between-class variability in reactive and, especially, proactive aggression, which justified class-level analysis, the majority of the variance of aggressive behavior was at the individual-level. This is a very common pattern in the school aggression literature, which may partly explain why it is not always easy to find significant predictors at the class-level. Finally, research on peer aggression at school has consistently identified the significant role of other class-level predictors, even within the realm of negative teacher-student relationships (e.g., Thornberg et al., 2018; Ten Bokkel et al., 2023). An additional, not necessarily alternative possibility for this lack of significant findings, therefore, may be that other class-level variables (e.g., class attitudes and norms) play a more central role compared to perceived relational teacher unfairness in differentiating class-level ratings of peer aggression, while teacher unfairness may demonstrate its negative impact more at the individual-level of analysis.

Regarding school satisfaction, instead, class-level analysis confirmed that there was a large degree of between-class variability in school satisfaction at T2 and that this variability was partially explained by class-level teacher unfairness. That is, not only individual students reported to be less satisfied if they felt to be treated unfairly by their teachers but, on average, school satisfaction toward the end of the school year was found to be significantly lower in classrooms where classmates shared a perception of unfair treatment. This effect was quite strong and robust against the control variables and is consistent with findings from studies with adult samples, mainly within organizations, showing that people satisfaction is associated not only with their individual perceptions of being treated fairly, but also with shared perceptions of a “fair context” (i.e., the average of individual perceptions of fairness within a group; e.g., Mossholder et al., 1998; Naumann and Bennett, 2000). This is an important novel contribution to the still limited literature on teacher relational unfairness within the classroom, showing for the first time a longitudinal negative effect of class-level teacher unfairness on students’ school satisfaction during one school year.

### 4.3 Limitations and implications

One limitation of this study is the relatively brief time span between the first and second waves. While the study made a significant contribution by examining both the individual-level and class-level impacts of perceived teacher unfairness over time, the conclusions drawn from our findings are limited to a period of approximately 6 months. Since we did not gather data over multiple years, we were unable to investigate how the relationships between the predictors and outcomes might evolve over a more extended period. It is recommended that future research expand beyond a single school year and potentially track adolescents over several years to gain a deeper understanding of these dynamics.

Second, all variables were evaluated using self-report questionnaires. It should be noted that self-report data may be influenced by social desirability bias, although it is often the most suitable or only option for researchers. In the context of this study, the primary focus was on the beliefs and perceptions of adolescents, which can only be conveyed by the individuals themselves. Moreover, relying solely on peer nominations or ratings to assess aggressive behavior may not always be reliable, as students may not accurately report their peers’ aggressive tendencies. In certain instances, individuals possess exclusive knowledge of specific aspects of their behavior, such as covert aggression. However, it is crucial to verify the findings of this study through longitudinal research that incorporates multiple sources of information regarding students’ aggressive behavior. Furthermore, including insights from teachers on their relationships with students, particularly in terms of fairness, could provide informative perspectives and enhance our understanding of classroom dynamics. Regarding teacher unfairness, a future line of research would be to compare students’ beliefs with ‘reality,’ to test whether different patterns of results emerge between students who believe that they are treated unfairly by their teacher, which is not true, and those who also have the same perception, but which corresponds to actual unfair treatment by the teacher.

Finally, in accordance with prior studies (Engels et al., 2016), students were asked to provide feedback on their overall experience with teachers in the classroom, rather than focusing on a specific teacher. This decision was made due to the fact that students interact with multiple teachers on a daily basis, making it impractical to gather individual feedback for each teacher. While this approach offers a broad understanding of how adolescents perceive their relationship with teachers, it is important to acknowledge that it may lack specificity. Consequently, this limits the ability to examine if certain teacher characteristics (such as sex, years of experience, or teaching hours per week) influence the connection between teacher unfairness and adolescent school adjustment. Moreover, it restricts the exploration of different patterns of teacher relationships, such as having one teacher who is fair and supportive while others are not, or having all teachers who are fair and supportive.

Despite the limitations of this study, the findings suggest that minimizing teacher unfairness is important for reducing student aggression and improving their school satisfaction. Effective conflict reduction strategies can help teachers recognize students’ good behavior and achievements, set high expectations, and build constructive relationships with individual students (Stipek and Miles, 2008). For example, teachers can negotiate behavioral contracts with students with whom they tend to experience the most conflict. These contracts should outline agreed-upon criteria for appropriate classroom behavior and interactions with the teacher and classmates (Bowman-Perrott et al., 2015). Additionally, teachers can use “connective instruction,” particularly interpersonal connectiveness, as a strategy to build positive relationships with their students (Martin and Dowson, 2009). The task at hand entails the active engagement of listening attentively to the perspectives expressed by students. It requires providing them with the opportunity to contribute to decisions that directly impact their lives. Moreover, it necessitates treating all students with equality, avoiding any form of bias, and affirming their worth. It also involves embracing and respecting their unique qualities and characteristics. Lastly, it entails maintaining a positive outlook and setting achievable goals for each student (Flink et al., 1990; Teven and McCroskey, 1997; Slade, 2001). Considering the moderating role of students’ gender, at least for some variables, tailored strategies should also be implemented that take into account the possible differential role of teacher unfairness for different gender groups.

Finally, schools should implement activities able to promote and increase a sense of school belonging which can help to mitigate the negative impact of negative teacher-student relationships on students’ school satisfaction. One promising strategy is to use “small learning communities” (Tillery et al., 2013). Briefly, these communities encompass the practice of organizing students into smaller networks within the broader school environment. These networks share common objectives and support systems, fostering a sense of connection and belonging among both students and adults. Consequently, educational institutions may contemplate implementing such initiatives to enhance the overall sense of community and affiliation within their schools.

## Author’s note

The data used in this study were extracted from a larger dataset of a longitudinal project examining the social-cognitive factors associated with aggressive behavior in adolescents. A portion of this dataset was previously utilized in another study on the moral predictors of aggressive behavior (Gini et al., 2022). Although the sample is the same, there is minimal overlap between the data used in the previous study and the current study, with only age, sex, and reactive and proactive aggression being common variables, serving different research purposes. The data on teacher unfairness, school satisfaction, and school stress have never been used in previously published manuscript. Moreover, the theoretical frameworks, the respective literatures, and the specific research questions of the two manuscripts are different.

## Data availability statement

The raw data supporting the conclusions of this article will be made available by the authors, without undue reservation.

## Ethics statement

The studies involving humans were approved by Ethics Committee for Psychological Research of the University of Padova. The studies were conducted in accordance with the local legislation and institutional requirements. Written informed consent for participation in this study was provided by the participants' legal guardians/next of kin.

## Author contributions

GG: Conceptualization, Data curation, Formal analysis, Methodology, Writing – original draft. FA: Data curation, Writing – review & editing. TP: Conceptualization, Writing – original draft.

## References

[ref1] Adams J. S. (1965). “Inequity in social exchange” in Advances in experimental social psychology. ed. Berkowitz L. (New York: Academic Press), 267–299.

[ref2] Arsenio W. Gold J. (2006). The effects of social injustice and inequality on children’s moral judgments and behavior: towards a theoretical model. Cogn. Dev. 21, 388–400. doi: 10.1016/j.cogdev.2006.06.005

[ref3] Baker J. A. (2006). Contributions of teacher–child relationships to positive school adjustment during elementary school. J. Sch. Psychol. 44, 211–229. doi: 10.1016/j.jsp.2006.02.002

[ref4] Bayram Özdemir S. Özdemir M. (2020). How do adolescents’ perceptions of relationships with teachers change during upper-secondary school years? J. Youth Adolesc. 49, 921–935. doi: 10.1007/s10964-019-01155-3, PMID: 31677083 PMC7105438

[ref5] Bowman-Perrott L. Burke M. D. de Marin S. Zhang N. Davis H. (2015). A meta-analysis of single-case research on behavior contracts: effects on behavioral and academic outcomes among children and youth. Behav. Modif. 39, 247–269. doi: 10.1177/014544551455138 25261083

[ref6] Bronfenbrenner U. (1979). The ecology of human development: experiments by nature and design. Cambridge, MA: Harvard University Press.

[ref7] Busching R. Krahé B. (2020). With a little help from their peers: the impact of classmates on adolescents’ development of prosocial behavior. J. Youth Adolesc. 49, 1849–1863. doi: 10.1007/s10964-020-01260-8, PMID: 32529342 PMC7423867

[ref8] Byrne D. G. Davenport S. C. Mazanov J. (2007). Profiles of adolescent stress: the development of the adolescent stress questionnaire (ASQ). J. Adolesc. 30, 393–416. doi: 10.1016/j.adolescence.2006.04.004, PMID: 16750846

[ref9] Card N. A. Stucky B. D. Sawalani G. M. Little T. D. (2008). Direct and indirect aggression during childhood and adolescence: a meta-analytic review of gender differences, intercorrelations, and relations to maladjustment. Child Dev. 79, 1185–1229. doi: 10.1111/j.1467-8624.2008.01184.x, PMID: 18826521

[ref10] Chen F. F. (2007). Sensitivity of goodness of fit indexes to lack of measurement invariance. Struct. Equ. Model. Multidiscip. J. 14, 464–504. doi: 10.1080/10705510701301834

[ref11] Cheung G. W. Rensvold R. B. (2002). Evaluating goodness of fit indexes for testing measurement invariance. Struct. Equ. Model. A Multidisc. 9, 233–255. doi: 10.1207/S15328007SEM0902_5

[ref12] Colquitt J. A. (2001). On the dimensionality of organizational justice: a construct validation of a measure. J. Appl. Psychol. 86, 386–400. doi: 10.1037/0021-9010.86.3.386, PMID: 11419799

[ref13] Crick N. R. Dodge K. A. (1994). A review and reformulation of social information-processing mechanisms in children's social adjustment. Psychol. Bull. 115, 74–101. doi: 10.1037/0033-2909.115.1.74

[ref14] Cropanzano R. Byrne Z. S. Bobocel D. R. Rupp D. E. (2001). Moral virtues, fairness heuristics, social entities, and other denizens of organizational justice. J. Vocat. Behav. 58, 164–209. doi: 10.1006/jvbe.2001.1791

[ref15] Danielsen A. G. Wiium N. Wilhelmsen B. U. Wold B. (2010). Perceived support provided teachers and classmates and students' self-reported academic initiative. J. Sch. Psychol. 48, 247–267. doi: 10.1016/j.jsp.2010.02.002, PMID: 20380949

[ref16] Enders C. K. Bandalos D. L. (2001). The relative performance of full information maximum likelihood estimation for missing data in structural equation models. Struct. Equ. Model. 8, 430–457. doi: 10.1207/S15328007SEM0803_5

[ref17] Engels M. C. Colpin H. Van Leeuwen K. Bijttebier P. Van Den Noortgate W. Claes S.. (2016). Behavioral engagement, peer status, and teacher–student relationships in adolescence: a longitudinal study on reciprocal influences. J. Youth Adolesc. 45, 1192–1207. doi: 10.1007/s10964-016-0414-5, PMID: 26759132

[ref18] Flink C. Boggiano A. K. Barrett M. (1990). Controlling teaching strategies: undermining children’s self-determination and performance. J. Pers. Soc. Psychol. 59, 916–924. doi: 10.1037/0022-3514.59.5.916

[ref19] Gilman R. Huebner E. S. Laughlin J. (2000). A first study of the multidimensional Students' life scale with adolescents. Soc. Indic. Res. 52, 135–160. doi: 10.1023/A:1007059227507

[ref20] Gini G. Marino C. Pozzoli T. Holt M. (2018). Associations between peer victimization, perceived teacher unfairness, and adolescents' adjustment and well-being. J. Sch. Psychol. 67, 56–68. doi: 10.1016/j.jsp.2017.09.005, PMID: 29571535

[ref21] Gini G. Pozzoli T. Bussey K. (2015). Moral disengagement moderates the link between psychopathic traits and aggressive behavior among early adolescents. Merrill-Palmer Q. 61, 51–67. doi: 10.13110/merrpalmquar1982.61.1.0051

[ref22] Gini G. Thornberg R. Bussey K. Angelini F. Pozzoli T. (2022). Longitudinal links of individual and collective morality with adolescents’ peer aggression. J. Youth Adolesc. 51, 524–539. doi: 10.1007/s10964-021-01518-9, PMID: 34661788 PMC8881436

[ref23] Hjern A. Alfven G. Östberg V. (2008). School stressors, psychological complaints and psychosomatic pain. Acta Paediatr. 97, 112–117. doi: 10.1111/j.1651-2227.2007.00585.x, PMID: 18076714

[ref24] Huebner E. S. (1994). Preliminary development and validation of a multidimensional life satisfaction scale for children. Psychol. Assess. 6, 149–158. doi: 10.1037/1040-3590.6.2.149

[ref25] James K. Bunch J. Clay-Warner J. (2015). Perceived injustice and school violence: an application of general strain theory. Youth Violence Juvenile Justice 13, 169–189. doi: 10.1177/1541204014521251

[ref26] Jungert T. Piroddi B. Thornberg R. (2016). Early adolescents’ motivations to defend victims in school bullying and their perceptions of student–teacher relationships: a self-determination theory approach. J. Adolesc. 53, 75–90. doi: 10.1016/j.adolescence.2016.09.001, PMID: 27654402

[ref27] Kazemi A. (2016). Examining the interplay of justice perceptions, motivation, and school achievement among secondary school students. Soc. Justice Res 29, 103–118. doi: 10.1007/s11211-016-0261-2

[ref28] Krause A. Smith J. D. (2022). Peer aggression and conflictual teacher-student relationships: a meta-analysis. Sch. Ment. Heal. 14, 306–327. doi: 10.1007/s12310-021-09483-1

[ref29] Lazarus R. S. (1966). Psychological stress and the coping process. New York: McGraw-Hill.

[ref30] Lenzi M. Vieno A. De Vogli R. Santinello M. Ottova V. Baška T.. (2013). Perceived teacher unfairness and headache in adolescence: a cross-national comparison. Int. J. Public Health 58, 227–235. doi: 10.1007/s00038-012-0345-1, PMID: 22314545

[ref31] Lenzi M. Vieno A. Gini G. Pozzoli T. Pastore M. Santinello M.. (2014). Perceived teacher unfairness, instrumental goals, and bullying behavior in early adolescence. J. Interpers. Violence 29, 1834–1849. doi: 10.1177/0886260513511694, PMID: 24366959

[ref32] Little T. D. Brauner J. Jones S. M. Nock M. K. Hawley P. H. (2003). Rethinking aggression: a typological examination of the functions of aggression. Merrill-Palmer Q. 49, 343–369. doi: 10.1353/mpq.2003.0014

[ref33] Löfstedt P. García-Moya I. Corell M. Paniagua C. Samdal O. Välimaa R.. (2020). School satisfaction and school pressure in the WHO European region and North America: an analysis of time trends (2002–2018) and patterns of co-occurrence in 32 countries. J. Adolesc. Health 66, S59–S69. doi: 10.1016/j.jadohealth.2020.03.007 32446610

[ref34] Longobardi C. Prino L. E. Marengo D. Settanni M. (2016). Student-teacher relationships as a protective factor for school adjustment during the transition from middle to high school. Front. Psychol. 7:1988. doi: 10.3389/fpsyg.2016.01988, PMID: 28066305 PMC5179523

[ref35] Longobardi C. Settanni M. Prino L. E. Fabris M. A. Marengo D. (2019). Students’ psychological adjustment in normative school transitions from kindergarten to high school: investigating the role of teacher-student relationship quality. Front. Psychol. 10:1238. doi: 10.3389/fpsyg.2019.01238, PMID: 31191415 PMC6548872

[ref36] Marengo D. Jungert T. Iotti N. O. Settanni M. Thornberg R. Longobardi C. (2018). Conflictual student–teacher relationship, emotional and behavioral problems, prosocial behavior, and their associations with bullies, victims, and bullies/victims. Educ. Psychol. 38, 1201–1217. doi: 10.1080/01443410.2018.1481199

[ref37] Martin A. J. Dowson M. (2009). Interpersonal relationships, motivation, engagement, and achievement: yields for theory, current issues, and educational practice. Rev. Educ. Res. 79, 327–365. doi: 10.3102/00346543083255

[ref38] MIUR (2019). MIUR – Ufficio di statistica: Gli alunni con cittadinanza non italiana (A.S. 2019/2020). Rome, Italy: MIUR.

[ref39] Mossholder K. W. Bennett N. Martin C. L. (1998). A multilevel analysis of procedural justice context. J. Organ. Behav. 19, 131–141. doi: 10.1002/(SICI)1099-1379(199803)19:2<131::AID-JOB878>3.0.CO;2-P

[ref40] Murdock T. B. (1999). The social context of risk: status and motivational predictors of alienation in middle school. J. Educ. Psychol. 91, 62–75. doi: 10.1037/0022-0663.91.1.62

[ref41] Muthén L. K. Muthén B. O. (1998–2017). Mplus user's guide (7th). Los Angeles, CA: Muthén & Muthén.

[ref42] Naumann S. E. Bennett N. (2000). A case for procedural justice climate: development and test of a multilevel model. Acad. Manag. J. 43, 881–889. doi: 10.5465/1556416

[ref43] Osterman K. F. (2000). Students' need for belonging in the school community. Rev. Educ. Res. 70, 323–367. doi: 10.3102/00346543070003323

[ref44] Polman H. De Castro B. O. Koops W. van Boxtel H. W. Merk W. W. (2007). A meta-analysis of the distinction between reactive and proactive aggression in children and adolescents. J. Abnorm. Child Psychol. 35, 522–535. doi: 10.1007/s10802-007-9109-4, PMID: 17340178

[ref45] Rasooli A. Zandi H. DeLuca C. (2019). Conceptualising fairness in classroom assessment: exploring the value of organizational justice theory. Assess. Educ. Princ. Policy Pract. 26, 584–611. doi: 10.1080/0969594X.2019.1593105

[ref46] Ren P. Wang Y. Liang Y. Li S. Wang Q. (2023). Bidirectional relationship between bullying victimization and functions of aggression in adolescents: the mediating effect of teacher justice. J. Adolesc. 95, 1245–1257. doi: 10.1002/jad.12198, PMID: 37244648

[ref47] Ripski M. B. Gregory A. (2009). Unfair, unsafe, and unwelcome: do high school students' perceptions of unfairness, hostility, and victimization in school predict engagement and achievement? J. Sch. Violence 8, 355–375. doi: 10.1080/15388220903132755

[ref48] Roeser R. W. Eccles J. Sameroff A. J. (1998). Academic and emotional functioning in early adolescence: longitudinal relations, patterns, and prediction by experience in middle school. Dev. Psychopathol. 10, 321–352. doi: 10.1017/S0954579498001631 9635227

[ref49] Sabbagh C. Resh N. (2016). Unfolding justice research in the realm of education. Soc. Justice Res 29, 1–13. doi: 10.1007/s11211-016-0262-1

[ref50] Salmivalli C. Voeten M. (2004). Connections between attitudes, group norms, and behaviour in bullying situations. Int. J. Behav. Dev. 28, 246–258. doi: 10.1080/01650250344000488

[ref51] Samdal O. Nutbeam B. Wold B. Kannas L. (1998). Achieving health and educational goals through schools—a study of the importance of school climate and the students' satisfaction with school. Health Educ. Res. Theory Pract. 13, 383–397. doi: 10.1093/her/13.3.383

[ref52] Santinello M. Vieno A. De Vogli R. (2009). Primary headache in Italian early adolescents: the role of perceived teacher unfairness. Headache 49, 366–374. doi: 10.1111/j.1526-4610.2008.01208.x, PMID: 18651853

[ref53] Santinello M. Vieno A. De Vogli R. (2011). Bullying in Italian schools: the role of perceived teacher unfairness. Eur. J. Psychol. Educ. 26, 235–246. doi: 10.1007/s10212-010-0050-5

[ref54] Skarlicki D. P. Folger R. (1997). Retaliation in the workplace: the roles of distributive, procedural, and interactional justice. J. Appl. Psychol. 82, 434–443. doi: 10.1037/0021-9010.82.3.434

[ref55] Slade M. (2001). Listening to boys. Boys Sch. Bull. 4, 10–18.

[ref56] Stipek D. Miles S. (2008). Effects of aggression on achievement: does conflict with the teacher make it worse? Child Dev. 79, 1721–1735. doi: 10.1111/j.1467-8624.2008.01221.x, PMID: 19037945

[ref57] Stipek D. Salmon J. M. Givvin K. B. Kazemi E. Saxe G. MacGyvers V. L. (1998). The value (and convergence) of practices suggested by motivation research and promoted by mathematics education reformers. J. Res. Math. Educ. 29, 465–488. doi: 10.2307/749862

[ref58] Swearer S. M. Hymel S. (2015). Understanding the psychology of bullying: moving toward a social-ecological diathesis–stress model. Am. Psychol. 70, 344–353. doi: 10.1037/a0038929, PMID: 25961315

[ref59] Szumski G. Smogorzewska J. Grygiel P. (2020). Attitudes of students toward people with disabilities, moral identity and inclusive education-a two-level analysis. Res. Dev. Disabil. 102:103685. doi: 10.1016/j.ridd.2020.103685, PMID: 32422515

[ref60] Ten Bokkel I. M. Roorda D. L. Maes M. Verschueren K. Colpin H. (2023). The role of affective teacher–student relationships in bullying and peer victimization: a multilevel meta-analysis. Sch. Psychol. Rev. 52, 110–129. doi: 10.1080/2372966X.2022.2029218

[ref61] Teven J. J. McCroskey J. C. (1997). The relationship of perceived teacher caring with student learning and teacher evaluation. Commun. Educ. 46, 1–9. doi: 10.1080/03634529709379069

[ref62] Thornberg T. Wänström L. Pozzoli T. Gini G. (2018). Victim prevalence in bullying and its association with teacher–student and student–student relationships and class moral disengagement: a class-level path analysis. Res. Pap. Educ. 33, 320–335. doi: 10.1080/02671522.2017.1302499

[ref63] Tillery A. D. Varjas K. Roach A. T. Kuperminc G. P. Meyers J. (2013). The importance of adult connections in adolescents' sense of school belonging: implications for schools and practitioners. J. Sch. Violence 12, 134–155. doi: 10.1080/15388220.2012.762518

[ref64] Torsheim T. Cavallo F. Levin K. A. Schnohr C. Mazur J. Niclasen B.. (2016). Psychometric validation of the revised family affluence scale: a latent variable approach. Child Indic. Res. 9, 771–784. doi: 10.1007/s12187-015-9339-x, PMID: 27489572 PMC4958120

[ref65] Tyler T. R. Lind E. A. (1992). A relational model of authority in groups. Adv. Exp. Soc. Psychol. 25, 115–192. doi: 10.1016/S0065-2601(08)60283-X

[ref66] Tyler T. R. Smith H. J. (1999). “Justice, social identity, and group processes” in The psychology of the social self. eds. Tyler T. R. Kramer R. M. John O. P. (Mahwah, New Jersey: Lawrence Erlbaum Associates), 223–264.

[ref67] Vieno A. Gini G. Santinello M. Lenzi M. Nation M. (2011). Violent behavior and unfairness in school: multilevel analysis of Italian schools. J. Community Psychol. 39, 534–550. doi: 10.1002/jcop.20450

[ref68] Wendorf C. A. Alexander S. (2005). The influence of individual- and class-level fairness-related perceptions on student satisfaction. Contemp. Educ. Psychol. 30, 190–206. doi: 10.1016/j.cedpsych.2004.07.003

